# Metagenomic analysis of blood microbiota alterations: insights into HIV progression and immune restoration

**DOI:** 10.3389/fcimb.2025.1619059

**Published:** 2025-10-20

**Authors:** Yingjie Chen, Rongqiu Zhang, Juan Wen, Jingyue Zhao, Jianmei Zhang

**Affiliations:** Department of Laboratory, Xiamen Center for Disease Control and Prevention, Xiamen, Fujian, China

**Keywords:** blood microbiota, metagenomic sequencing, HIV infection, antiretroviral therapy, correlation analysis

## Abstract

**Introduction:**

Emerging evidence suggests that the blood microbiome may influence the progression of HIV infection and immune restoration. This study aims to comprehensively characterize blood microbiota alterations associated with HIV infection and antiretroviral therapy (ART), and to evaluate their potential as microbial indicators for assessing infection status and immune restoration.

**Methods:**

We recruited 91 participants, including 31 treatment-naïve HIV-infected individuals, 30 ART-treated individuals with undetectable viral loads, and 30 healthy controls. Blood samples were collected for metagenomic sequencing and immunological profiling.

**Results:**

HIV infection profoundly disrupted blood microbiota diversity and composition, with a marked reduction in α-diversity and enrichment of opportunistic pathogens such as *Pseudomonas aeruginosa*, *Acinetobacter baumannii* and *Stenotrophomonas maltophilia*, alongside depletion of beneficial taxa like *Bifidobacterium longum*. ART partially restored microbial diversity but did not fully reestablish a healthy microbiota. Correlation analysis revealed that *Acinetobacter pittii*, *Xanthomonas campestris* and *Diaphorobacter nitroreducens* were significantly associated with viral load, suggesting their potential role in HIV progression. Additionally, after ART, *Acinetobacter junii* and *Pseudomonas putida* were significantly correlated with the CD4/CD8 ratio, indicating their potential role in immune restoration.

**Discussion:**

These findings provide new insights into the interactions between blood microbiota and HIV progression. The identified blood microbiota may serve as potential indicators for evaluating HIV infection status and treatment efficacy, offering a basis for microbial-based diagnostic and therapeutic strategies.

## 1 Introduction

Human immunodeficiency virus (HIV) remains a major global public health challenge, characterized by persistent CD4+ T cell depletion, impaired mucosal barrier function and chronic immune activation (Kaslow et al., 2024). As the immune system becomes progressively compromised, impaired CD4+ T-cell regeneration and elevated CD8+ T-cell counts further contribute to immune dysregulation and systemic inflammation. The CD4/CD8 ratio is a key immunological indicator of HIV disease progression and prognosis, with a low ratio often indicative of chronic immune activation and immune failure (Obeagu et al., 2023). Antiretroviral therapy (ART) effectively suppresses viral load, slows CD4+ T cell depletion, and partially restores the CD4/CD8 ratio, thereby delaying the progression to acquired immunodeficiency syndrome (AIDS) (Sterne et al., 2009).

HIV infection disrupts mucosal immunity, leading to microbial translocation, which exacerbates chronic inflammation and disease progression. Gut-associated lymphoid tissue (GALT) plays a crucial role in maintaining gastrointestinal mucosal integrity (Sandgaard et al., 2014). GALT harbors the majority of the body’s CD4+ T lymphocytes and serves as a key site for HIV replication, dissemination, and reservoir formation (Rosado-Sánchez et al., 2016). During early HIV-1 infection, massive depletion of CD4+ T cells occurs in GALT, accompanied by significant structural and functional disruption of the mucosal barrier (Taisheng et al., 2011). Consequently, microbial translocation occurs, allowing bacterial components or live microorganisms to enter the bloodstream, triggering systemic immune activation and inflammation (Lao et al., 2022). Numerous studies have investigated gut microbiota composition in HIV-infected individuals, revealing significant alterations compared to healthy controls. Specifically, HIV-infected individuals exhibit an increase in pro-inflammatory and potentially pathogenic bacteria, coupled with a reduction in beneficial microbes (Sun et al., 2024).

The concept of a “sterile” bloodstream has been challenged in recent years. The primary source of microorganisms detected in blood is thought to be the intestinal and oral microbiota via atopobiosis (Dragomanova et al., 2025). Increasing evidence supports the presence of a circulating blood microbiome and its potential relevance to disease (Amar et al., 2011, Amar et al., 2013; Puri et al., 2018; Ciocan et al., 2021), and blood microbial DNA has been proposed as a potential biomarker in certain specific disease contexts (Dragomanova et al., 2025). Several studies directly comparing blood and gut microbial communities further highlight their distinct but related nature. For example, Tan (Tan et al., 2023) profiled microbial DNA in the blood of 9,770 healthy individuals and identified 117 species, with only partial overlap with gut taxa, which suggests that blood signatures may reflect transient microbial translocation rather than a stable microbiome. Similarly, paired blood–stool analyses have shown that blood microbial profiles are taxonomically distinct from gut communities, yet remain influenced by gut permeability and microbial translocation (Shah et al., 2021; Scarsella et al., 2023; Zhai et al., 2024). Together, these findings underscore the value of profiling microbial DNA in blood as a complementary window into host–microbiome interactions, while not interchangeable with gut microbiota profiling.

However, the role of blood microbiota in HIV infection and immune restoration remains poorly understood. A recent study (Nganou-Makamdop et al., 2021) found that in HIV-infected individuals, the diversity and composition of translocated microorganisms in plasma influence the host inflammatory state, thereby modulating T cell reconstitution outcomes following ART initiation. Additionally, Sergio Serrano-Villar et al (Serrano-Villar et al., 2021)observed that patients with better immune restoration after ART (as indicated by a significant improvement in CD4/CD8 ratio) exhibited specific bacterial signatures in plasma, such as *Actinobacteria* and *Lactobacillales*, which correlated with inflammatory markers (e.g., sCD14, sCD163, and CRP), suggesting a potential role in immune modulation. Similarly, Xiaoyan Guo et al (Guo et al., 2023)reported that microbial species enriched in treatment-naïve patients (TNs) and immune non-responders (INRs) showed a positive correlation with HIV DNA and RNA, whereas species enriched in healthy controls and immune responders (IRs) were negatively correlated. These findings strongly suggest that the immune status of HIV-infected individuals is likely associated with blood microbiome alterations. However, further research is needed to elucidate the precise role of blood microbiota in HIV progression and immune restoration following ART.

In this study, we performed metagenomic sequencing to comprehensively characterize the blood microbiome of HIV-infected individuals before and after ART. We further explored the association between blood microbial taxa and key immunological indicators, including viral load and T cell subsets, to assess the potential of blood microbiota as indicators of HIV infection status and immune restoration. Our findings provide new insights into the interactions between blood microbiota and HIV progression and offer a basis for microbial-based diagnostic and therapeutic strategies.

## 2 Materials and methods

### 2.1 Study population and sample collection

This study included 91 participants, categorized into three groups: 31 newly diagnosed HIV-infected individuals who had not received antiretroviral therapy, 30 HIV-infected individuals undergoing ART with a viral load below the detection limit (20 copies/mL), and 30 healthy controls. All ART-treated participants received the standard first-line regimen recommended by national guidelines (TDF + 3TC + EFV). Healthy controls were recruited from the Xiamen Center for Disease Control and Prevention (CDC), were HIV antibody-negative, and had no infectious or systemic diseases. To minimize potential confounding effects on the microbiome, all participants who had used antibiotics or probiotics within the month prior to enrollment and sampling were excluded from the study.

Venous blood samples were collected from all participants in duplicate. One sample was used for plasma separation and stored at -80°C for DNA extraction, while the other was used for T-cell analysis and viral load quantification. The time from blood draw to centrifugation did not exceed 2 hours. Demographic and clinical characteristics, including name, sex, age, infection route, and immunological parameters, were recorded. The HIV infection duration was calculated as the interval between the date of confirmed HIV diagnosis and the sampling date, while the ART duration was calculated as the interval between ART initiation and the sampling date.

### 2.2 Viral load and T-cell analysis

HIV viral load was measured using the COBAS AmpliPrep Nucleic Acid Extraction System and the COBAS TaqMan48 Analyzer (Roche). T cell subpopulation analyses were performed using four-color fluorescent labeling (PC5, FITC, ECD, PE) and analyzed on a CYTOMICS FC 500 flow cytometer (Beckman Coulter). All procedures were performed according to the manufacturer’s protocols.

### 2.3 DNA extraction, library preparation, and sequencing

Plasma samples stored at -80°C were thawed, and 1.5 mL plasma from each sample was used for DNA extraction. DNA was extracted from blood samples in a biosafety cabinet using the QIAamp^®^ UCP Pathogen Mini Kit (Qiagen), following the manufacturer’s instructions. Library preparation was conducted using the MGISEQ-200RS high-throughput sequencing kit (FCL PE100). The library concentration was quantified using a Qubit 3.0 fluorometer (Invitrogen). Three blank controls were included and underwent DNA extraction and library preparation in parallel with the samples. Sequencing was performed on an MGISEQ-200 platform (BGI).

### 2.4 Sequence quality control and taxonomic analysis

Raw sequencing reads were processed using SOAPnuke (version 2.1.7) for quality filtering and adapter removal. Low-complexity filtering was applied to remove reads with a quality score < 15 or > 0.2 fraction of low-quality bases. Host sequences were eliminated by mapping against the human reference genome (hg19) using Snap-aligner (version 2.0.1) with parameters “-h 5000 -t 70”. No duplicate read filtering was applied. The remaining sequences were classified taxonomically using Kraken2 (version 2.1.2) with a confidence threshold of 0.6 and a minimum-hit requirement of 2, and species abundance was estimated with Bracken (version 2.5).

### 2.5 Microbial diversity analysis

Alpha diversity indices, including Shannon, Simpson, and evenness indices, were calculated using the vegan package (version 2.6.8) in R. Beta diversity was assessed using Bray-Curtis and a compositionality-aware Aitchison (CLR-Euclidean) analysis, both visualized via principal coordinate analysis (PCoA). Group differences were tested by PERMANOVA (999 permutations) and dispersion homogeneity by PERMDISP.

### 2.6 Microbial composition analysis

Relative abundance was defined as the proportion of reads assigned to each taxon out of the total microbial reads per sample. At the family and genus levels, the top 15 most abundant taxa were identified, with remaining species classified as “Others” to ensure a total relative abundance of 100%. At the species level, relative abundances were compared across groups using Kruskal–Wallis tests; taxa with *p* < 0.05 were considered significant. Among significant taxa, the 30 species with the highest total relative abundance were displayed in a heatmap (asterisks indicate significance). Taxonomic composition was further illustrated using stacked bar plots generated with the ggalluvial package (version 0.12.5).

### 2.7 Differential taxa analysis

Low-abundance taxa detected in fewer than 20% of samples were excluded. Unique species present in only one group were reported with frequency and abundance, while species found in multiple groups were analyzed using the Mann-Whitney U test to identify significantly different taxa (*p* < 0.05).

### 2.8 LEfSe analysis

Linear discriminant analysis effect size (LEfSe) analysis was conducted using the microeco package (version 1.11.1). The analysis was performed with a minimum sample requirement of 10, a subsampling ratio of 66.67%, and 30 resampling iterations. Multiple testing correction was applied using the Benjamini-Hochberg method, with a significance threshold of alpha = 0.01 and an LDA score cutoff of > 3.

### 2.9 Spearman correlation analysis

Spearman rank correlations were computed between species relative abundance and clinical immunological indicators using the rcorr (Hmisc), and are reported as ρ together with raw ρ values and Benjamini-Hochberg FDR-adjusted *q* values. *q* < 0.05 was considered statistically significant, *p* < 0.05 but *q* ≥ 0.05 was considered nominal associations. Heatmaps display correlations filtered at *p* < 0.05 for visualization.

### 2.10 Statistical analysis

Categorical variables were analyzed using Fisher’s exact test. Continuous variables were tested for normality using the Shapiro-Wilk test and for homogeneity of variance using Levene’s test. If data met normality and homogeneity assumptions, one-way ANOVA with Tukey’s HSD *post hoc* test was applied, with results presented as Mean ± SD. Otherwise, the Kruskal-Wallis test followed by Dunn’s test was used, with results reported as Median [IQR]. Statistical significance was defined as: *p* < 0.001(***), *p* < 0.01 (**), *p* < 0.05 (*), p > 0.05 (*ns*, not significant). All statistical analyses were conducted in RStudio (version 4.3.2).

## 3 Results

### 3.1 Basic characteristics of the study population

A total of 91 participants were enrolled in this study, including 30 healthy individuals (NHS group), 31 untreated HIV-infected individuals (HIV group), and 30 ART-treated individuals with undetectable viral loads (ART group). There was no statistically significant difference in age among the three groups (*p* = 0.081), but there was a significant difference in gender distribution due to the higher proportion of men who have sex with men (MSM) among HIV-infected patients (*p* < 0.001). Due to the lack of data on immunological indicators in the NHS group, this study only compared the blood immunological indicators between the HIV and ART groups. The results showed that the CD4+ cell count, CD4+ cell percentage and CD4+/CD8+ ratio were significantly lower in the HIV group than in the ART group, while the CD8+ cell count and CD8+ cell percentage were significantly higher than in the ART group. These immunological changes were closely associated with HIV infection and ART status. The clinical information and immunological characteristics of the study participants are summarized in **Table 1**.

**Table 1:** Characteristics of HIV-infected and uninfected patients.

Characteristics	NHS group	HIV group	ART group	P value
Subjects (n)	30	31	30	
Age (yr)	35.77 ± 6.48	29 [26.5 - 41.5]	35 [31 - 50]	8.10E-02
Gender, n (%)				4.03E-07
Male	11	29	26	
Female	19	2	4	
Infection duration (days)	–	37(33–37)	213(201–226)	1.52E-11
ART duration (days)	–	–	188(172–204)	
T cell count (cells/μL)				
CD3+	–	1535 ± 565	1588 ± 514	7.04E-01
CD4+	–	266 [159.5 - 351]	614 [416.25 - 722]	3.03E-07
CD8+	–	1063 [909 - 1455.5]	843 [722.25 - 900]	9.61E-03
T cell percent (%)				
CD3+	–	70.4 ± 13.7	65.8 ± 9.54	1.33E-01
CD4+	–	11.8 [8.4 - 14.65]	22.8 [18.27 - 30.2]	5.36E-08
CD8+	–	57.6 [43.65 - 62.65]	35.8 [32.88 - 41.2]	1.46E-05
CD4+/CD8+ (%)	–	0.2 [0.15 -0.3]	0.7 [0.4 -0.9]	5.68E-08
Viral load (copies/mL)	–	104000 [58400 - 250000]	< 20^a^	

^a^HIV viral load values below detection limit are recorded as ‘<20’.

### 3.2 Impact of HIV infection and ART on blood microbial diversity

**Figure 1:**
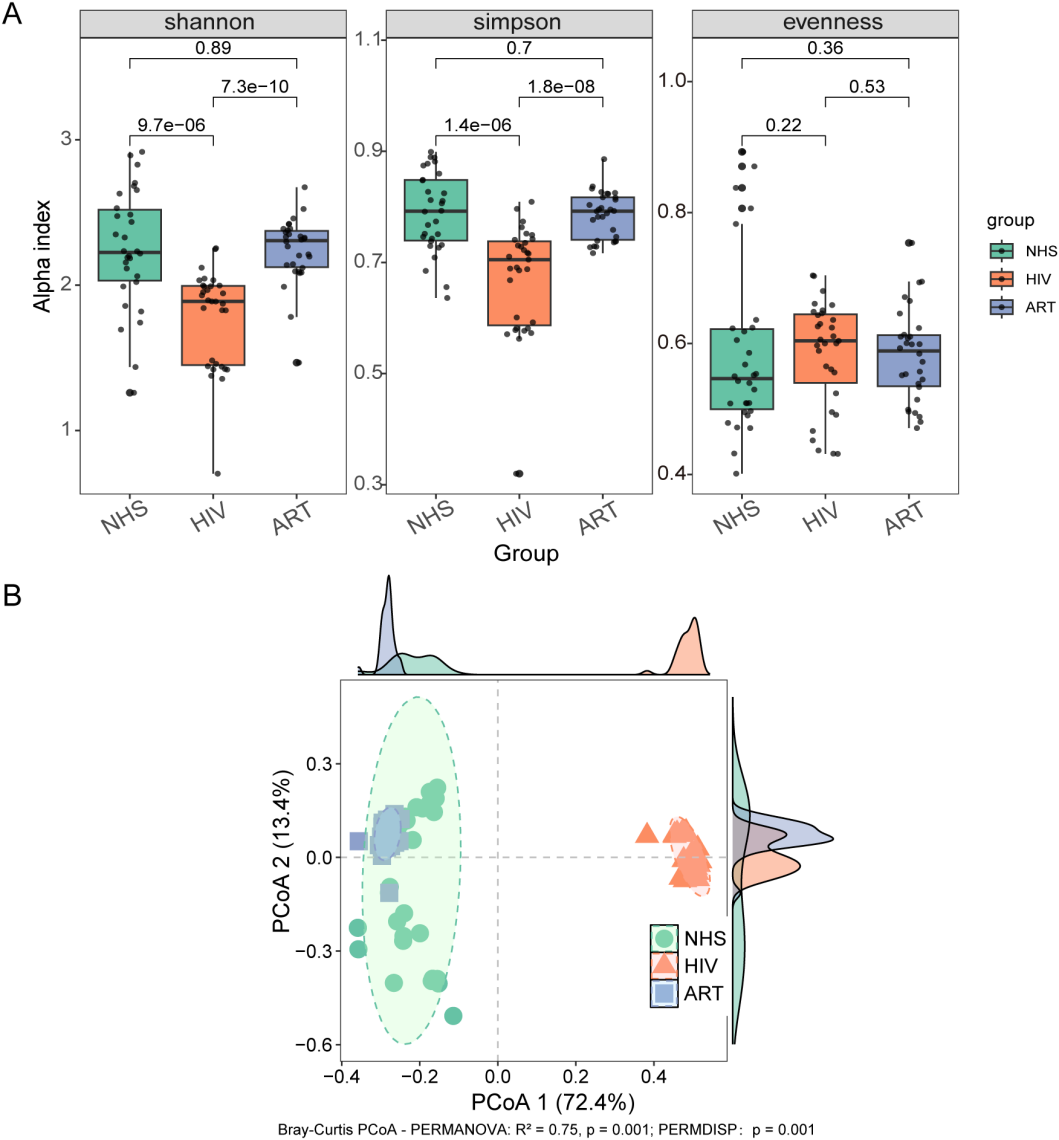
Bacterial microbiota structure in blood. **(A)** Alpha diversity analysis showing differences in Shannon index, Simpson index, and evenness index among the NHS, HIV, and ART groups. **(B)** PCoA based on Bray-Curtis distance. The *R²* value represents the proportion of variation explained by the grouping factor, with higher values indicating a stronger effect of grouping on microbial composition.

HIV infection significantly affected the α-diversity of the blood microbiota. Compared with the NHS group, the HIV group exhibited a marked decrease in microbial richness (Shannon index: *p* = 9.7e-06; Simpson index: *p* = 1.4e-06), while microbial evenness (Pielou’s evenness) showed no significant difference (*p* > 0.05, **Figure 1A**.

Beta diversity based on Bray-Curtis distance revealed clear separation in microbial community composition among groups (**Figure 1B**). Pairwise PERMANOVA showed marked compositional differences between NHS and HIV (*R²* = 0.705, *p* < 0.001) and between HIV and ART (*R²* = 0.858, *p* < 0.001), while ART remained distinct from NHS (*R²* = 0.139, *p* < 0.001) despite partially restoring microbial diversity. PERMDISP analysis further indicated significant differences in within-group dispersion across cohorts (*p* = 0.001), suggesting that both centroid shifts and dispersion effects contributed to the observed group separations. In addition to Bray-Curtis, an Aitchison (CLR-Euclidean) analysis also revealed significant group separations (PERMANOVA: *R²* = 0.33, *p* = 0.001; PERMDISP: *p* = 0.001; **Supplementary Figure S1**), confirming that the observed differences are robust under a compositional framework. Taken together, these analyses indicate that HIV infection is associated with marked alterations in blood microbiota composition, and that ART shifts the profiles toward but does not fully restore the NHS state.

### 3.3 Impact of HIV infection and ART on blood microbial composition

**Figure 2:**
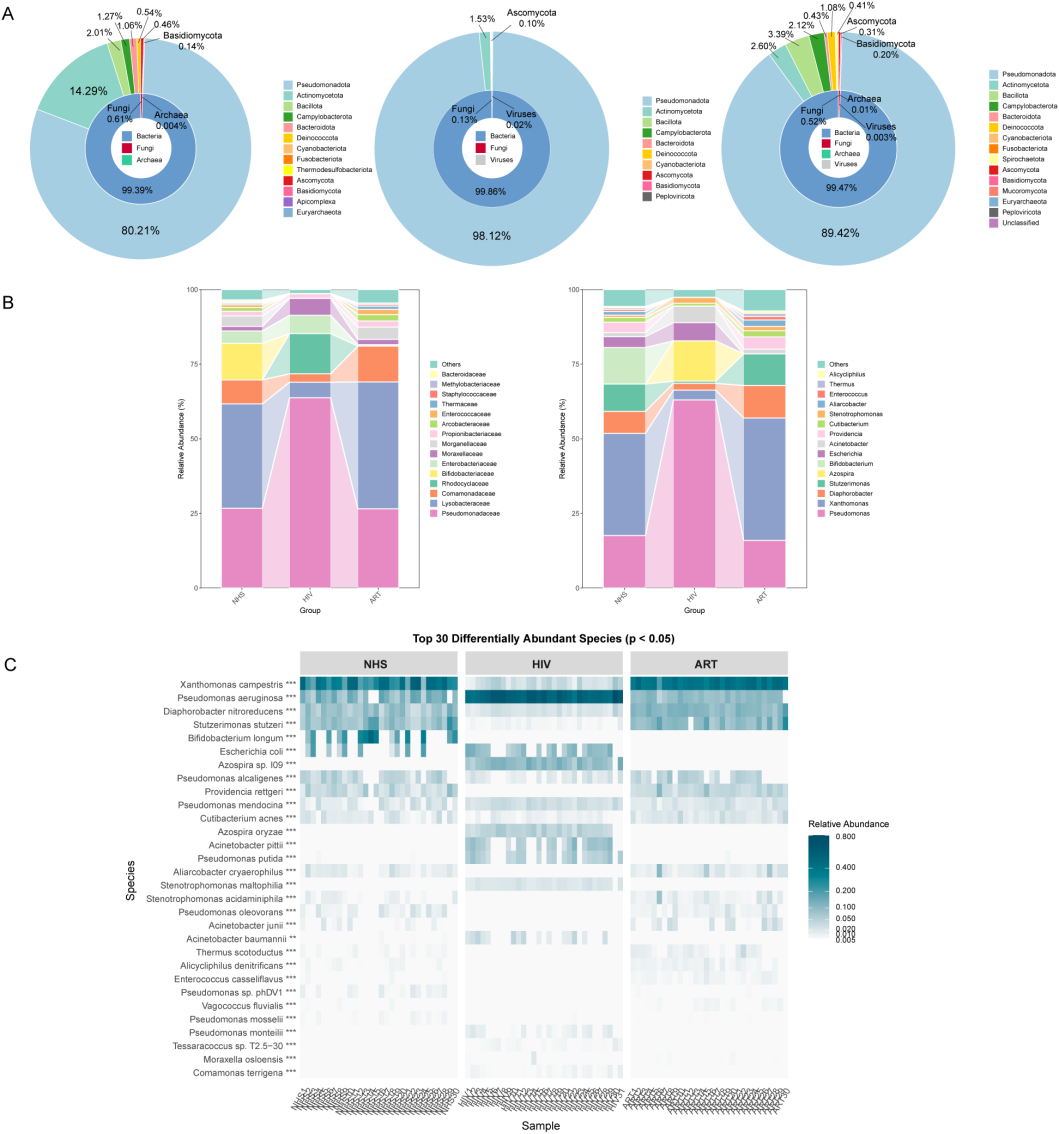
Composition of blood microbiota at different taxonomic levels. **(A)** Relative abundance of microbiota at the kingdom and phylum levels. From left to right, the three sunburst plots represent the NHS, HIV, and ART groups. The inner ring of each plot represents the kingdom-level composition, while the outer ring shows the phylum-level composition. **(B)** Relative abundance at the family level (left) and genus level (right), presented as a stacked bar chart. **(C)** Heatmap of the top 30 differentially abundant species (*p* < 0.05) across the three groups, with color intensity representing relative abundance.

Significant differences in blood microbial composition were observed among the groups. Sunburst plot analysis revealed that bacteria were the dominant taxa across all three groups, followed by fungi, with *Pseudomonadota* as the most abundant phylum (**Figure 2A**). The NHS group exhibited a more balanced microbial community, with higher abundance of *Actinomycetota* and *Bacillota.* The HIV group showed a significant increase in *Pseudomonadota* abundance, whereas *Actinomycetota* was markedly reduced. After ART, *Pseudomonadota* abundance decreased and *Actinomycetota* increased compared with the HIV group, but their levels did not fully reach those of the NHS group, indicating only partial restoration.

Analyses at the family and genus levels (**Figure 2B**) showed that the microbial composition of the NHS group was more diverse, mainly including *Pseudomonadaceae* (*Pseudomonas* and *Stutzerimonas*), *Lysobacteraceae* (*Xanthomonas*), *Comamonadaceae* (*Diaphorobacter*), *Enterobacteriaceae* (*Escherichia*), and *Bifidobacteriaceae* (*Bifidobacterium*). In the HIV group, *Pseudomonas* became the dominant genus, accounting for 63.04% of the total microbiota, accompanied by a significant increase in *Escherichia* (*Enterobacteriaceae*) and *Acinetobacter* (*Moraxellaceae*). Conversely, *Xanthomonas*, *Diaphorobacter*, *Stutzerimonas*, and *Bifidobacterium* were significantly reduced or even absent. In addition, *Azospira* sp. was newly detected in the HIV group, reflecting microbial niche alterations under immune suppression. Following ART, the composition shifted toward that of the NHS group, with increased abundances of *Xanthomonas*, *Stutzerimonas*, and *Diaphorobacter*, but *Bifidobacterium* markedly lower, further indicating only partial restoration rather than complete recovery.

Screening of the top 30 significantly different species at the species level (**Figure 2C**) showed that HIV infection significantly increased the relative abundance of *Pseudomonas aeruginosa* and *Pseudomonas putida*, whereas *Xanthomonas campestris*, *Diaphorobacter nitroreducens*, and *Stutzerimonas stutzeri* were significantly reduced, likely due to microbial dysbiosis and competitive disadvantage. In addition, *Azospira* sp. 109, *Azospira oryzae*, and *Acinetobacter pittii* were newly detected in the HIV group, possibly due to intestinal barrier dysfunction and the translocation of environmental microbes into the bloodstream. Although ART partially reversed these changes, beneficial microbes such as *Bifidobacterium longum* remained at very low levels (0.01%), again supporting that ART only partially rather than fully restored the blood microbial composition.

### 3.4 Identification of potential microbial indicators associated with HIV infection

**Figure 3:**
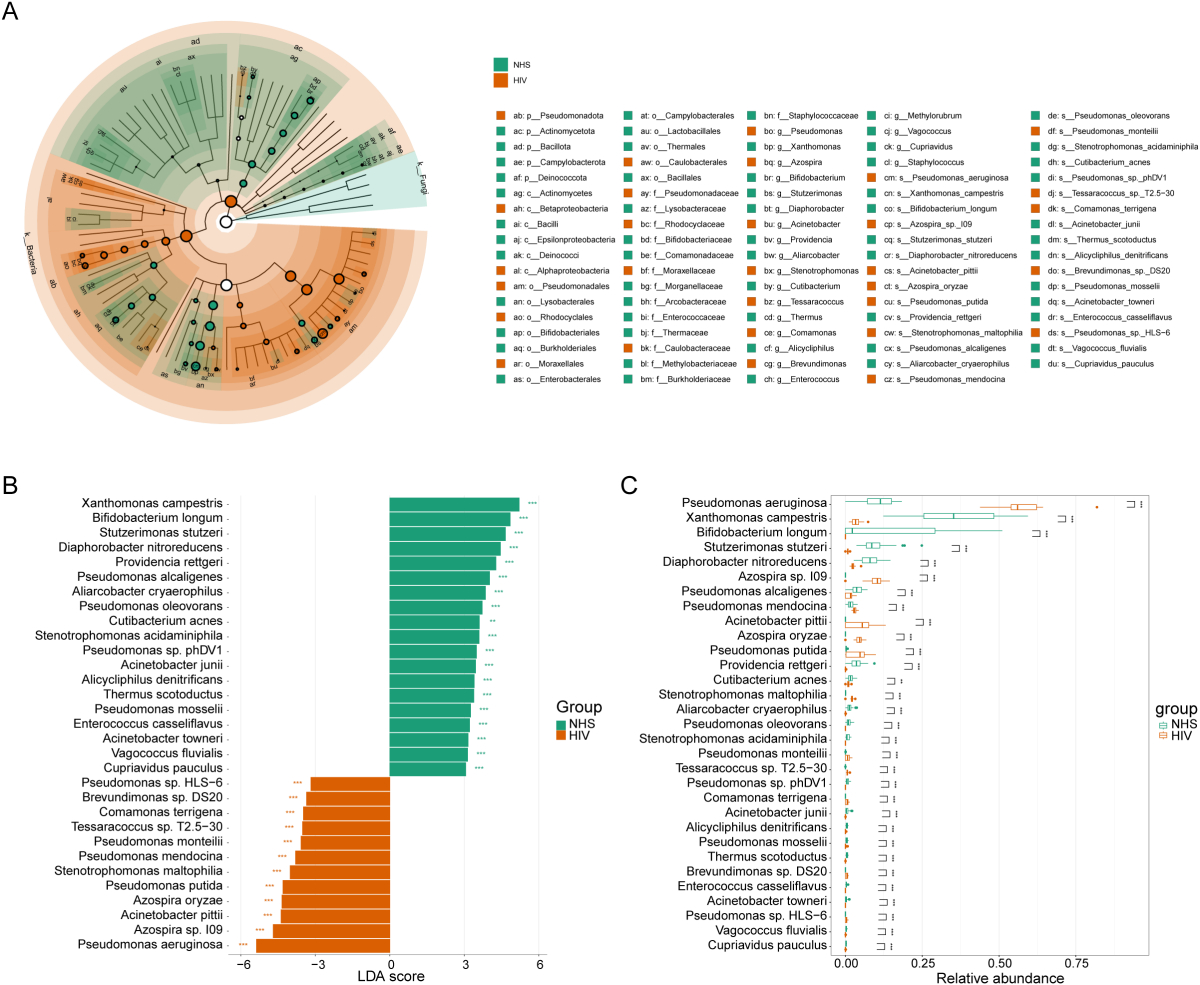
Differentially abundant species between NHS and HIV groups. **(A)** Cladogram illustrating taxonomic differences from the phylum to species levels between NHS and HIV groups. Node size is proportional to the LDA score, and colors indicate species significantly enriched in each group. **(B)** LDA score plot ranking differentially abundant species at the species level. A higher LDA score indicates a greater contribution to group differentiation. **(C)** Boxplots comparing the relative abundance differences of 31 species identified in **(B)**.

To identify potential microbial indicators distinguishing the NHS and HIV groups, 87 differentially abundant species were first screened out using the Mann-Whitney U-test (**Supplementary Table S1**). LEfSe analysis further confirmed 89 taxa with significant differences between the two groups (LDA > 3, *p* <0.01) (**Figure 3A**), among which 30 taxa (e.g., *Pseudomonas* and *Azospira*) were enriched in the HIV group, while 59 taxa (e.g., *Bifidobacterium* and *Xanthomonas*) were enriched in the NHS group. Further screening identified 31 species with higher LDA scores (**Figure 3B**), among which *Pseudomonas aeruginosa*, *Azospira* sp. *109*, *Acinetobacter pittii*, *Azospira oryzae*, and *Pseudomonas putida* were significantly more abundant in the HIV group, whereas *Xanthomonas campestris*, *Stutzerimonas stutzeri*, *Diaphorobacter nitroreducens*, *Pseudomonas alcaligenes*, and *Providencia rettgeri* were more abundant in the NHS group (**Figure 3C**). These findings suggest that HIV infection significantly alters the composition of the blood microbiota, particularly the relative abundances of *Pseudomonas* spp. and *Xanthomonas* spp., which may be associated with host immune function and have potential applications in distinguishing HIV-infected individuals from healthy individuals.

**Figure 4:**
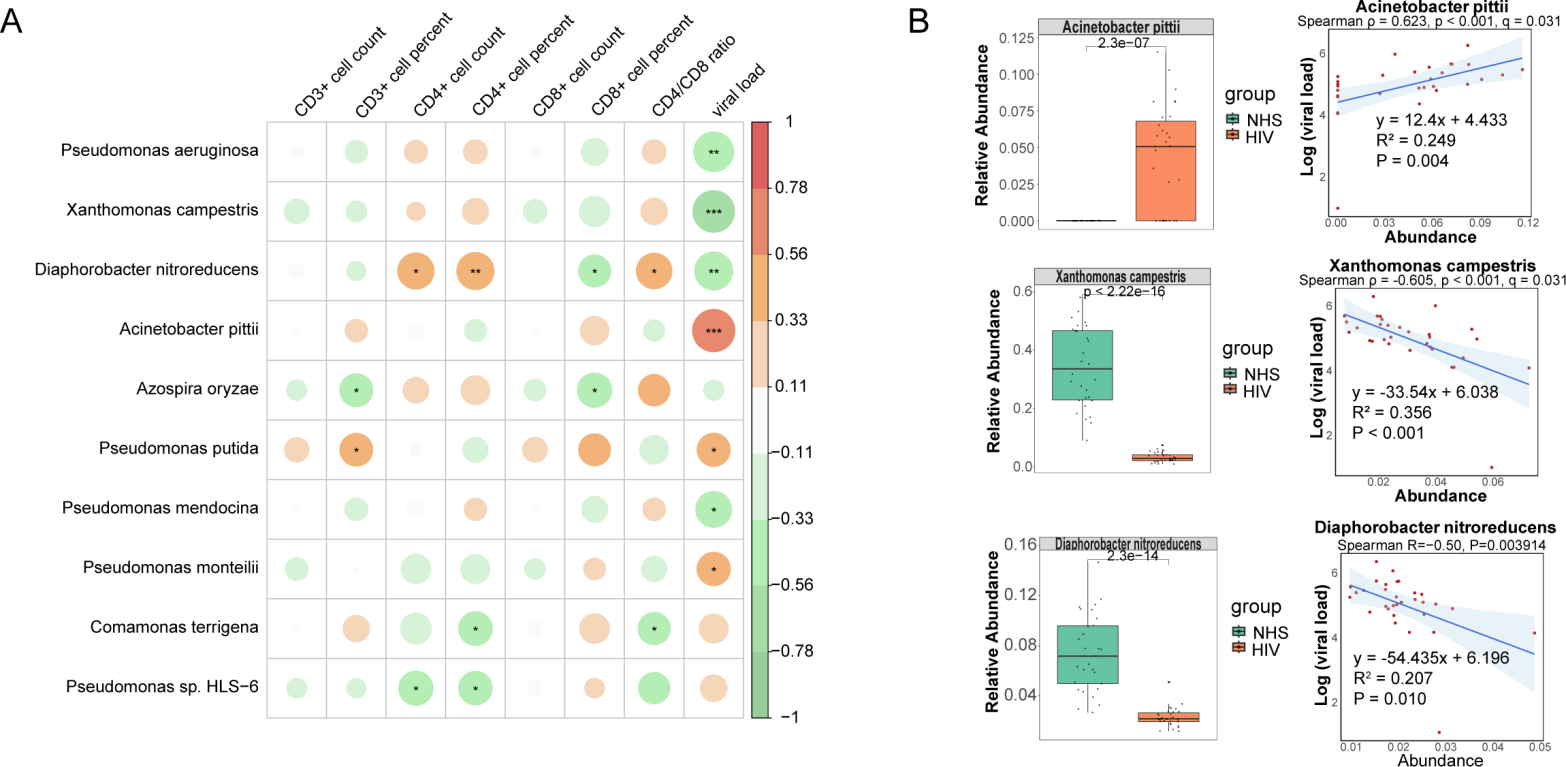
Correlations between species and immunological indicators within the HIV group. **(A)** Spearman correlation analysis between species abundances and immunological indicators. **(B)** The group-wise abundance differences and linear regression analysis of species significantly correlated with immunological indicators.

To further investigate the potential relationship between these 31 significantly different species and immunological indicators, and to assess their potential role in reflecting HIV infection severity, we conducted Spearman correlation analysis between these species and eight immunological indicators in the HIV group. Ten candidate species showed associations at *p* < 0.05 (**Figure 4A**). Among them, *Acinetobacter pittii* (ρ = 0.623, *p* < 0.001, q = 0.031) and *Xanthomonas campestris* (ρ = –0.605, *p* < 0.001, *q* = 0.031) remained significant after FDR correction, both showing strong correlations with viral load. In contrast, additional taxa such as *Pseudomonas aeruginosa* (ρ = –0.549, *p* = 0.001, *q* = 0.093) and *Diaphorobacter nitroreducens* (ρ = –0.503, *p* = 0.004, *q* = 0.196) exhibited only nominal negative correlations with viral load. The Mann-Whitney U test further validated these abundance differences (**Figure 4B**), which showed that *Acinetobacter pittii* was significantly increased in the HIV group, consistent with its positive correlation with viral load, while *Xanthomonas campestris* and *Diaphorobacter nitroreducens* were significantly reduced in the HIV group, consistent with their observed negative correlation with viral load. Additionally, we found that *Acinetobacter pittii*, *Xanthomonas campestris*, and *Diaphorobacter nitroreducens* exhibited significant linear correlations with viral load (**Figure 4B**). These findings suggest that the abundance of these species may serve as potential microbial indicators for distinguishing HIV-infected individuals from healthy controls and reflecting HIV infection severity, providing a potential reference for assessing HIV infection status. However, while *Pseudomonas aeruginosa* and *Pseudomonas mendocina* were also associated with viral load, their abundance trends between the NHS and HIV groups did not exactly as expected, their roles as indicators of the severity of HIV infection need to be further investigated.

### 3.5 Identification of potential microbial indicators associated with immune restoration

**Figure 5:**
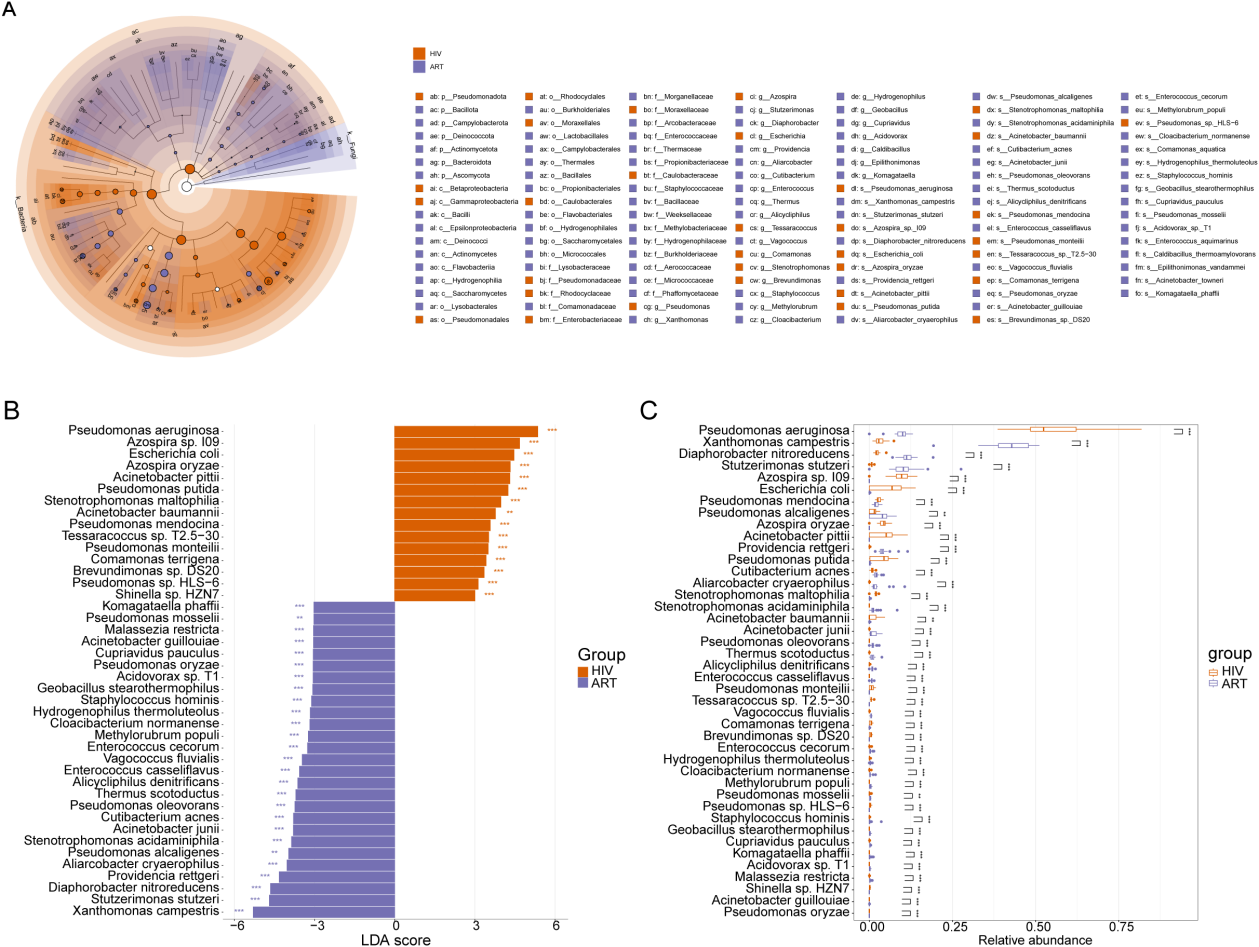
Differentially abundant species between the HIV and ART groups. **(A)** Cladogram illustrating taxonomic differences from phylum to species levels between the HIV and ART groups. Node size is proportional to the LDA score, and colors indicate species significantly enriched in each group. **(B)** LDA score plot ranking differentially abundant species at the species level. A higher LDA score indicates a greater contribution to group differentiation. **(C)** Boxplots comparing the relative abundances of the 43 species identified in **(B)**.

To investigate the impact of ART on blood microbiota composition and host immune function recovery, as well as to identify potential microbial indicators associated with immune restoration, we firstly screened 65 species with significant differences in abundance between the HIV group and the ART group using the Mann-Whitney U-test (**Supplementary Table S2**). LEfSe analysis further identified 124 taxa with significant differences between the two groups (LDA > 3, *p* < 0.01, **Figure 5A**), among which 34 taxa were enriched in the HIV group, including *Pseudomonadota*, *Pseudomonadales*, *Pseudomonadaceae*, and *Pseudomonas*, while 59 taxa were enriched in the ART group, including *Comamonadaceae*, *Burkholderiales*, *Lysobacteraceae*, and *Diaphorobacter*. Further screening identified 43 species with high LDA scores (**Figure 5B**), among which *Pseudomonas aeruginosa, Azospira* sp.*109*, and *Escherichia coli* were significantly more abundant in the HIV group, while *Xanthomonas campestris, Diaphorobacter nitroreducens*, and *Stutzerimonas stutzeri* were significantly more abundant in the ART group (**Figure 5C**).

**Figure 6:**
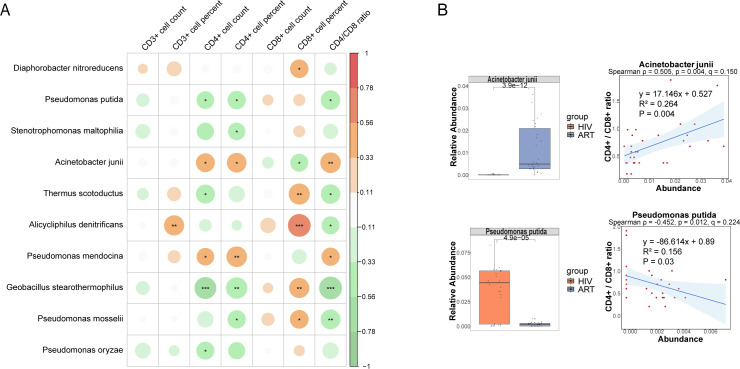
Correlations between species and immunological indicators within the ART group. **(A)** Spearman correlation analysis between species abundances and immunological indicators. **(B)** The group-wise abundance differences and linear regression analysis of species significantly correlated with immunological indicators.

To assess whether these differentially abundant species were associated with immune function indicators in the ART group, we performed Spearman correlation analysis between the 43 species and seven immune indicators. Ten species showed associations at *p* < 0.05. Among them, *Geobacillus stearothermophilus* (*ρ* = –0.626, *p* < 0.001, *q* = 0.030) was significantly negatively correlated with the CD4/CD8 ratio, and *Alicycliphilus denitrificans* (*ρ* = 0.621, *p* < 0.001, *q* = 0.030) was significantly correlated with CD8+ T cell percentage, both remained significant after FDR correction. In contrast, additional taxa such as *Acinetobacter junii* (*ρ* = 0.505, *p* = 0.004, *q* = 0.150) and *Pseudomonas putida* (*ρ* = –0.452, *p* = 0.012, *q* = 0.224) exhibited only nominal correlations with the CD4/CD8 ratio (**Figure 6A**). Mann-Whitney U tests revealed that *Acinetobacter junii* was significantly more abundant in the ART group, consistent with its positive correlation with immune function indicators. Conversely, *Pseudomonas putida* exhibited lower relative abundance in the ART group, consistent with its negative correlation with the CD4/CD8 ratio (**Figure 6B**). Additionally, a significant linear correlation was observed between the abundance of *Acinetobacter junii* and *Pseudomonas putida* and the CD4/CD8 ratio in the ART group, suggesting that these species could serve as potential microbial indicators for assessing immune restoration. However, although *Geobacillus stearothermophilus, Pseudomonas mosselii, Diaphorobacter nitroreducens*, and *Thermus scotoductus* were associated with immune indicators, their abundance trends between the HIV and ART groups did not fully align with expectations, require further investigation.

## 4 Discussion

HIV infection can impair GALT structure and immune function, facilitating the translocation of microorganisms and their metabolites into the bloodstream, thereby influencing disease progression (Brenchley et al., 2006; Estes et al., 2010; Sandler and Douek, 2012; Zevin et al., 2016). In this study, metagenomic sequencing of blood microbiota DNA in HIV-infected individuals and healthy controls revealed distinct microbial alterations associated with HIV infection and assessed the impact of ART on these changes. Furthermore, we explored the potential of blood microbiota as microbial indicators for evaluating HIV infection status and treatment efficacy, providing novel insights for precision diagnosis and therapeutic strategies. It is important to note that our metagenomic approach detects microbial DNA rather than viable bloodstream microbiota. Microbial DNA detected in whole blood may derive from circulating or blood cell–associated microbes, or from microbial cell-free DNA (cfmDNA) released by degraded or secreting cells (Pietrzak et al., 2023). Therefore, the detection of microbial DNA should not be interpreted as evidence of live bacteria in the bloodstream.

Our metagenomic sequencing results demonstrated that HIV-infected individuals exhibit unique microbial signatures in the bloodstream. In contrast to prior studies suggesting that HIV-induced gut barrier dysfunction leads to increased microbial translocation and higher *α*-diversity (Serrano-Villar et al., 2021), we observed a significant decrease in blood microbiota *α*-diversity following HIV infection, as evidenced by reductions in Shannon and Simpson indices. This reduction may be attributed to the depletion of beneficial bacteria resulting from GALT dysfunction, accompanied by the overgrowth of opportunistic pathogens. Differential species abundance analyses further support this hypothesis, showing a significant reduction in beneficial bacteria such as *Bifidobacterium longum* and a marked increase in opportunistic pathogens, including *Pseudomonas aeruginosa*, *Acinetobacter baumannii*, and *Stenotrophomonas maltophilia*. These findings align with the immunosuppressive state associated with HIV infection, suggesting that GALT damage may contribute to blood microbiota dysbiosis.

ART partially restored the *α*-diversity of blood microbiota in HIV-infected individuals, consistent with previous findings by (Peng, 2022), which suggest that ART may contribute to GALT repair and the re-establishment of microbial homeostasis. However, PCoA analysis showed that the microbiota composition in the ART group had not fully returned to a healthy state, with key beneficial bacteria such as *Bifidobacterium* remaining at low levels. One plausible upstream factor is heterogeneity in the latent HIV reservoir: larger reservoirs may sustain low-grade immune activation and delay mucosal repair despite plasma viral suppression, thereby impeding the re-expansion of commensals such as *Bifidobacterium*. In addition, because our measurements derive from blood, the signals reflect compartment-specific translocation rather than gut composition per se; partial barrier repair under ART may preferentially restrict commensal leakage into the circulation. Together, these considerations suggest that while ART effectively suppresses HIV replication, its capacity to fully restore microbial homeostasis remains limited, underscoring the potential value of adjunctive microbiota-targeted strategies to complement ART.

Notably, this study identified several opportunistic pathogens significantly enriched in the HIV group, such as *Pseudomonas aeruginosa* and *Acinetobacter baumannii*. *Pseudomonas aeruginosa*, a well-known opportunistic pathogen, has been reported to cause severe infections, including bacteremia, urinary tract infections, and hospital-acquired pneumonia, particularly in immunocompromised individuals (Swathirajan et al., 2018). Moreover, it may exacerbate HIV-related inflammatory responses (Yuan et al., 2019). The risk of *Pseudomonas aeruginosa* infection is closely associated with CD4 T-cell levels (Sorvillo et al., 2001), with a significantly higher incidence observed in patients with CD4 counts below 200 cells/μL (Huang and Crothers, 2010). Similarly, *Acinetobacter baumannii*, a multidrug-resistant pathogen, poses a serious threat to immunocompromised individuals and those receiving broad-spectrum antibiotic treatments (Yang et al., 2018). Our findings align with those of Sergio Serrano-Villar et al (Serrano-Villar et al., 2021)., who also detected opportunistic pathogens in the bloodstream of untreated HIV-infected individuals, further supporting the hypothesis that HIV-associated immune dysfunction may facilitate pathogen translocation.

Additionally, we detected *Azospira* sp. *109* and *Azospira oryzae* in the blood of HIV-infected individuals, which were not detected in the NHS and ART groups. These species typically classified as environmental bacteria, which have been shown to invade and colonize the gut under immunosuppressed conditions (Yang et al., 2016), subsequently translocating into the bloodstream and causing infections. *Stenotrophomonas maltophilia*, another environmental Gram-negative bacterium, has recently been recognized as an emerging opportunistic pathogen in HIV-infected individuals (Calza et al., 2003), capable of causing severe infections in immunocompromised patients (Mojica et al., 2022), such as those with cystic fibrosis or cancer. In the present study, significant enrichment of *Stenotrophomonas maltophilia* in the HIV group was also observed, which provides important reference for co-infection studies in HIV-infected individuals in China. These findings suggest that HIV infection may facilitate the invasion of specific pathogens and increase the risk of opportunistic infections, whereas ART, to some extent, reduces the abundance of these pathogens in the bloodstream, thereby mitigating infection risk.

Beyond investigating microbiota alterations, we explored the role of blood microbiota in assessing HIV infection status and ART efficacy. LEfSe analysis revealed that *Xanthomonas campestris, Bifidobacterium longum*, and *Stutzerimonas stutzeri* were enriched in the NHS group, potentially indicating their association with immune homeostasis in the healthy state. Conversely, HIV infection may impair immune function, disrupt microbial barriers, and facilitate the proliferation of pathogenic species such as *Pseudomonas aeruginosa* in the bloodstream. Correlation and abundance trend analyses further demonstrated that specific microorganisms, such as *Acinetobacter pittii, Xanthomonas campestris*, and *Diaphorobacter nitroreducens*, were significantly associated with viral load, suggesting that blood microbiota may play an important role in HIV disease progression and could serve as potential microbial indicators of infection severity. For instance, *Xanthomonas campestris* was enriched in the NHS group and negatively correlated with viral load, suggesting a possible protective role, whereas *Acinetobacter pittii* was significantly enriched in the HIV group and positively correlated with viral load, indicating its potential involvement in HIV-related immune dysfunction. In the ART group, some specific microorganisms (e.g., *Xanthomonas campestris* and *Diaphorobacter nitroreducens*) were re-enriched as viral load was suppressed and partial immune restoration occurred. Moreover, *Acinetobacter junii* and *Pseudomonas putida* were significantly correlated with the CD4/CD8 ratio, suggesting their potential role in ART-mediated immune restoration. *Acinetobacter junii* was significantly positively correlated with the CD4/CD8 ratio and may play a synergistic role in the immune restoration process, whereas *Pseudomonas putida* was significantly negatively correlated with the CD4/CD8 ratio and significantly decreased following ART, suggesting its possible association with chronic inflammation or immune activation in HIV-infected individuals. These observations align with findings from Serrano-Villar et al., who also reported correlations between specific blood microbes and inflammatory or immune activation markers in HIV-infected individuals (Serrano-Villar et al., 2021). However, some species, such as *Pseudomonas aeruginosa* and *Geobacillus stearothermophilus*, did not exhibit abundance trends exactly as expected, despite being statistically correlated with immune indicators. This phenomenon may be attributed to the limited sample size or inter-individual variability, including extreme values. Therefore, future studies should include larger cohorts and longitudinal data to validate the biological significance of these microbial changes.

In contrast to previous studies primarily focused on gut microbiota, this study provides a novel perspective by investigating blood microbiota alterations in HIV infection and ART treatment. By integrating immune parameters, we systematically evaluated the potential of blood microbiota as microbial indicators for predicting HIV infection status and ART treatment efficacy. However, this study has certain limitations. First, the case-control, cross-sectional design precludes causal inference and does not allow assessment of longitudinal outcomes such as opportunistic infections or patient survival. Future longitudinal cohorts with clinical follow-up will be needed to clarify links between CD4/CD8 decline, microbial alterations, and long-term prognosis. Second, we did not collect direct indicators of intestinal barrier integrity (e.g., zonulin, I-FABP) or cytokine and soluble inflammation markers (e.g., LPS, sCD14, sCD163). As mucosal disruption and systemic inflammation are central to HIV pathogenesis, future studies will incorporate validated permeability and inflammation markers to examine these mechanisms more directly. Third, only blood samples were analyzed, precluding within-cohort gut–blood comparisons; paired stool–blood sampling with prospective recording of dietary and medication variables will be important to link gut sources to circulating microbial signals. Fourth, HIV reservoir size (e.g., total or integrated HIV DNA, intact proviral DNA) was not quantified, limiting our ability to test whether reservoir burden contributes to the incomplete recovery of beneficial taxa such as *Bifidobacterium* under ART. Finally, the relatively high proportion of MSM participants and the modest sample size may introduce selection bias. Moreover, unmeasured variables such as smoking, co-infections, and lifestyle factors could also have contributed to the observed variability. In addition, PERMDISP analysis indicated differences in within-group dispersion, suggesting that part of the variance may reflect heterogeneity rather than shifts in group centroids alone. Future studies with larger, more balanced cohorts, stricter batch control, and expanded negative controls will be needed to confirm the robustness of these findings.

## Data Availability

The datasets presented in this study have been deposited in the NCBI Sequence Read Archive (SRA) under BioProject accession number PRJNA1225636. The data are publicly available at https://www.ncbi.nlm.nih.gov/bioproject/PRJNA1225636.
