# Supplementary figures and images for: Metagenomic analysis of blood microbiota alterations: insights into HIV progression and immune restoration
Source: Front Cell Infect Microbiol. 2025 Oct 20;15:1619059. doi: 10.3389/fcimb.2025.1619059 (PMC12580212; doi:10.3389/fcimb.2025.1619059)

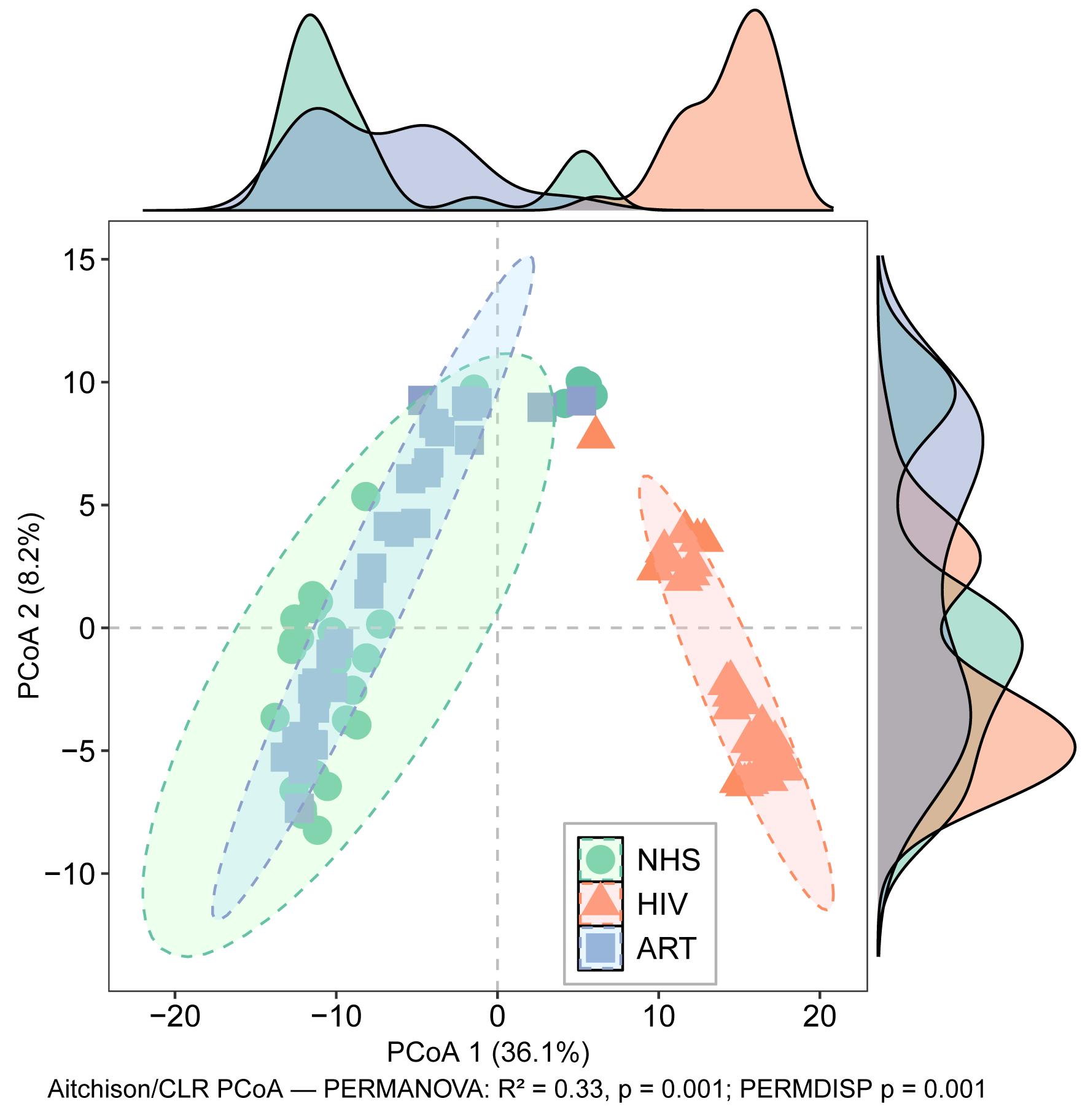

Supplement: Supplementary file 1 [file Image1.tif]
